# The Commissioners in Lunacy's Report on Bethlem Hospital

**Published:** 1853-01-01

**Authors:** R. W. F. Lutwidge


### THE COMMISSIONERS IN LUNACY'S REPORT ON BETHLEM HOSPITAL.

TO THE RIGHT HON. SIR GEORGE GREY, BART., &C.

February 7, 1852.

Sir,—We, the Commissioners in Lunacy, report that, by virtue of your order dated 13th June, 1851, authorizing two or more of us to visit Bethlem Hospital, four members of this board visited that institution on the 7th July last, and saw all or the greater number of the patients resident therein, and made a general inspection of the wards, rooms, and exercise-grounds appropriated to their use.

Upon that occasion the visiting commissioners were accompanied by the matron during their passage through the wards used by the female patients, and by the resident apothecary during their inspection of the entire establishment. After having visited the wards, and before leaving the hospital, the Commissioners made various inquiries of the resident apothecary, of the matron, of Dr. Monro, one of the visiting physicians of the institution (who called at the hospital during the commissioners' visit), and two of the nurses of the establishment, in reference to certain points to which their attention had been directed, and also as to the care and attention generally bestowed on the patients; and the answers given to these inquiries disclosed facts that appeared to the Commissioners to deserve a more minute scrutiny.

It will be in your recollection that our application to you for an order to visit this hospital, was founded mainly on the case of Miss A. M., respecting which information had been laid before us, together with certain matters of complaint, into the accuracy of which, as a board superintending the general treatment of lunatic patients in England, we felt it to be our duty to inquire. Subsequently to the visit made on the 7th of July, it became known that an inquiry was pending before this Board into some matters connected with Bethlem Hospital, and thereupon certain evidence was volunteered to be laid before us, which we did not think it right to reject; the more especially as it appeared to bear materially on the general object of our inquiry.

We therefore, at different times, summoned before us and examined various witnesses, and required the production of several documents, tending to show, not only the special treatment adopted in reference to particular patients, but also the character of the general arrangement and supervision of the hospital, in which the treatment of the cases brought especially under our cognizance was immediately or indirectly involved.

It is right to state that every facility was afforded by the governors of Bethlem Hospital, to enable us to pursue our inquiries.

Before submitting the result of our inquiry, it will be convenient to advert to the leading characteristics of the institution.

The net income of the estate applicable to the purposes of the charity appears from the general account of 1850-51 to be 17,400*l.* per annum, which is exclusive of about 3,000*l.* per annum paid by Government for the maintenance and care of criminal patients, making a total income of about 20,400*l.*

The hospital is united with the hospital of Bridewell, both of which, with their revenues, were granted to the Corporation of the City of London, and are now nominally under the general direction of a body of governors at present exceeding 300 in number. Out of this general body is formed a managing committee, consisting (besides the president and treasurer) of forty-two governors, and this committee subdivides itself into various sub-committees. Amongst others, a sub-committee of seven members is summoned by rotation to attend weekly at Bethlem Hospital. This sub-committee, however, is open to the whole body of governors.

It is customary for four, five, or six governors to attend the meeting of the Bethlem sub-committee, the duties of which are to determine on the admission and discharge of patients; "to examine those in the house;" to audit bills, &c.; and for general purposes.

Sometimes it is said that the treasurer, who, in the absence of the president, is the standing chairman, is alone present, and constitutes in his own person this sub-committee. The sub-committee go through the wards of the hospital once a month, but never at night.

The sub-committee report to the general committee of forty-two governors, who meet at Bridewell, and by whom the proceedings of the sub-committee are required to be confirmed.

The principal officers are the president, the treasurer (who ranks next to the president), two visiting physicians, one consulting surgeon, the resident apothecary, a steward, a matron, and various subordinate officers.

The treasurer is a responsible officer, to whom the execution of various important duties is committed. In consideration of his services to the institution and to the Bridewell Hospital, he has the free use of a furnished house at Bridewell, in which he resides. His powers appear to be very extensive. His duties, as stated by himself, are to superintend the affairs of the hospital,

"both as to its attendants and details." He has no power, however, he states, to interfere with the medical officers, or matron, except as an individual member of the board, or to rescind any of the existing rules. Nevertheless, he says that in a case of emergency he takes upon himself to rescind a rule, reporting such interference on his part immediately to the committee. On one occasion, indeed, he seems to have assigned to the matron, in our opinion very injudiciously, the very important power of classifying, employing, and generally of managing and arranging the female patients, without reporting such alteration to the committee, or obtaining their sanction thereto.

The treasurer generally presides at the weekly meetings, when reports from the steward and medical officers are submitted to the board, and the case-book (so far only as relates to patients who are from time to time discharged) is laid before, and signed by him. The hiring and discharge of attendants also rest with him. In the case of hiring male attendants and servants, he acts on the recommendation of the resident apothecary and steward respectively, and on hiring female attendants, on the recommendation of the matron. His power of dismissal appears to be absolute. The diet of the patients, together with much of the internal economy and comfort of the institution, appears to be under his influence.

It does not appear that any periodical visits to the wards are comprehended in the list of the treasurer's duties, but he does visit them about once a fortnight, and sometimes at uncertain times, but never at night.

Practically, the treasurer is the principal authority at the hospital, holding the position of a managing director, and the officers and others resident therein appear to be in a great measure under his control.

The visits of the treasurer and other members of the sub-committee are, for the most part, made at times when the attendants may expect and be prepared for them; very rarely, if ever, at night, and after the patients are in bed; and their attention on such occasions appears in general to be principally directed to the state of the wards, in regard to cleanliness, ventilation, and warmth, and to seeing that the patients are tranquil and orderly, and the attendants are in their proper places.

It is to be regretted, that the visitors on these inspections are invariably accompanied by an attendant, so that the patients have no separate opportunities of making complaints; and further, that no detailed entry or report recording the fact of the visit, and the particular matters observed, as calling for reprehension, inquiry, or amendment, is made or preserved in a written form.

The internal good order of the establishment, however, and the general comfort and well-being of the patients, in ordinary circumstances, must depend mainly on the degree of vigilance with which the attendants are superintended in the performance of their daily duties by the resident officers of the hospital.

The duties of the visiting physicians consist in each of them visiting the hospital four days a week, one of these days being on Friday, when the sub-committee assemble. Upon these visits, each physician generally sees the patients placed under his own care, or such of them as require medical treatment, but not those under the care of his colleague.

The precise duration of these visits cannot be easily ascertained; the physicians themselves estimating that they respectively last two or three hours, and sometimes only one hour, whilst another estimate fixes them at about one hour each. They are evidently of no long duration, for we understand that the limited attendance of one of the visiting physicians has already been made the subject of complaint by one of the governors of the institution.

On reference to the duties of the physicians, as set forth in the printed rules and orders, they appear to be (*inter alia*) to inform the committee of any neglect or abuse, and to suggest reformation; to examine and prescribe for the patients; to visit every part of the hospital, and to record their visits; and to sign the case-book, which (according to the rules and orders) is to contain the particulars of each case, as taken by the physician of the week, with an account of the remedies prescribed, and the result of such treatment.

How far the visiting physicians have made themselves acquainted with neglects and abuses, and have suggested remedies, we have no means of ascertaining, except in the several cases hereafter specially noticed. But it does not appear that they prescribe for all their patients, or that they visit all parts of the hospital, or that they sign any such case-books as are required to be kept by the rules and orders.

The surgeon's duties are mainly to attend in those cases only where a patient has any disorder requiring his assistance, and to view the bodies of patients after death, and leave a statement respecting them with the resident apothecary. It does not appear that he is called on to attend patients in ordinary or simple surgical cases, which are left to the care of the resident apothecary.

The duties of the resident apothecary (amongst many other things) are: 1st, To enter in a register the particulars of each case as taken by the physicians, with a weekly record of the bodily and mental health of every curable patient, and of any change from time to time in their condition, together with the effects of all medicines administered. 2nd, To visit the patients and wards daily, and to keep a record of such visits, and to report to the physicians any alteration which he may make in the treatment of patients. 3rd, To make occasional visits to the wards after the patients are in their sleeping-rooms, and to report such visits, with his observations thereon. And, 4th, To regulate, in the absence of the physicians, the classification, employment, amusements, and general management of the patients.

According to the accompanying evidence of the resident apothecary, by far the greater portion of the medical treatment of the patients appears, in practice, to devolve upon him, and thus his own peculiar duties are neglected; for it is manifest, from the whole of his testimony, that the medical and other registers and records of the hospital, which form so very important a feature in this officer's duties, and which, if perfect, would afford such material evidence as to the actual care and treatment bestowed upon the patients whilst in the hospital, are generally either very imperfectly kept or totally neglected. His night visits to the wards appear rare, and his visits to the female wards are very rare; and his powers and duties of classification, &c., conferred on him by the rules, have, so far as they relate to the females, been recently transferred to the matron by the verbal direction of the treasurer.

It seems unnecessary for the purpose of this report to advert to the duties of the steward, further than to state, that besides having the care of the stores, &c., he formerly had the control over the male attendants, who are now, however, properly placed, by some verbal regulation of the treasurer, under the management of the resident medical officer.

In regard to the matron, she, according to the rules and orders, is directed (amongst other things) to keep a record of all instances of restraint for the inspection of the medical officers, and also "to see that the linen and sheets of the patients are regularly changed as they ought to be," and to take an account of the linen, and to see that it is returned and properly washed. These are amongst the duties entrusted to her, in addition to the power of classifying and employing the female patients, lately transferred to her by the treasurer, as already referred to, and which she now exercises quite independently of the medical officers of the institution. It is also, according to her statement, a part of her duty to look to the personal comforts of the patients.

Although the matron makes daily visits to the female wards, and keeps written notes of anything particular that she may observe therein, she makes no report to any medical or other officer on the subject, it not being, she says, part of her duty to do so. Either from some misapprehension on her part, or from some alteration in the rules, of which we are not cognizant, she does not make any report on the subject of restraint, that report being now made by the nurses directly to the resident apothecary. Adverting to her duties to see that the linen of the patients is properly changed, washed, and returned, and that it is her duty to look to the personal comforts of the patients, it might be inferred, that the fact of as many as fifteen female patients sleeping naked in the basement-ward must have become known to her; but this she assures us was not the case—a fact that can only be attributable to her imperfect and not very frequent visits to this part of the hospital, and to the fact of her never seeing these patients after their being placed in their beds at night.

We have only further to remark, that the jurisdiction of the matron ought never to be allowed to interfere with, much less to supersede, the authority of the medical officer in anything that relates to the management, classification, or employment of the patients, whether male or female.

The attendants at Bethlem to whom the more immediate charge of the patients is committed, are sufficient in point of numbers, but the supervision exercised over them is evidently lax and superficial; and we can perceive that this laxity has produced its natural effect, by introducing among them a corresponding laxity of discipline.

Amongst other things, there is much reason to fear that practices have prevailed in some of the wards, which indicate the existence of great harshness and violence on the part of some of the attendants, and are in themselves utterly unjustifiable. We allude more particularly to the practice alleged to have been resorted to of laying down the females, who were dirty, on the stone floor of the gallery, naked, and washing their persons with cold water and a mop; to the brutal means which are said to have been sometimes employed for controlling and overpowering refractory patients; and to the violent and offensive mode in which food appears to have been forced upon some of those who obstinately refused to take it voluntarily.

To what extent these abuses may have prevailed in the hospital in former times can now be only matter of conjecture; but the fact of their having existed, and that at no remote period, seems sufficiently clear.

The patients of the hospital are distinguished into three classes—viz., those on the *curable*, those on the *incurable*, and those on the *criminal* lists respectively, of which the first is the most numerous and important.

In order to be admissible on the curable list, the rules of the hospital require that the attack of insanity shall not have been of more than twelve months' duration, and shall not be complicated with "paralysis, epilepsy, or any other disease of such a kind as to require the attendance of a nurse, or to threaten the speedy dissolution of life." In practice, if any such disease supervenes during residence in the hospital, the patient is sent away uncured, and in any event, whether recovered or not, he is discharged at the end of a twelvemonth, except in certain cases in which a few months' extension of the term of the residence, for the benefit of further treatment, is allowed.

The cases on the curable list may therefore be described as picked cases, that is to say, cases in which the mental disorder is of recent origin, and not combined with serious bodily illness, and presents the fairest prospect of recovery.

The average number of patients on the curable list at any one time is about 210; the average number admitted in the course of the year is about 315. In the course of the last year no fewer than 344 of such cases were received.

The patients on the incurable list consist of persons who have been formerly on the curable list, and have been discharged not cured.

These patients (who are not subject to the rules affecting the selection, discharge, and removal of curable patients) remain, with very rare exceptions, in the hospital during their lives. The average number on the incurable list may be taken at rather less than 80. The number admitted in the course of a year is on the average not more than 2.

The daily number of patients on the criminal list amounts on an average to little more than 100. The number admitted in the course of the year is on the average about 21. They are placed in a separate ward, and never mix with the other patients. They stand also on a totally different footing in regard to their admission and discharge, a matter which, in their case, is under the control of the Home Office; and as their wards constitute an entirely distinct department, the cost of which is defrayed by the Government, it cannot be regarded as a part of the charitable foundation of the hospital.

The total number of cases daily under treatment upon the average of the five years ending the 31st December, 1850, was 396, of whom 210 were on the curable, 80 on the incurable, and 106 on the criminal list.

The number of new cases annually admitted according to the same average was 367, of whom 316 were on the curable, 2 only on the incurable, and 21 on the criminal list. The total number of cases treated in the hospital in the course of the same five years was 1783 on the curable, 99 on the incurable, and 203 on the criminal list; giving an aggregate of not less than 2085 separate cases under treatment on the three lists during that period. The number of patients of all cases under treatment in the course of the year 1850 was 770.

With regard to classification, the patients of each sex, exclusive of the criminals, are, according to the general rules, directed to be distributed into five classes, and are placed in corresponding wards; the first class comprising dirty and refractory patients; the second, patients newly admitted, and who are in a state of probation; the third and fourth, patients in different stages of convalescence; and the fifth, incurable patients.

This mode of distribution is, in the main, carried out in practice. The refractory and dirty patients occupy what is termed No. 1, or the refractory ward, which is situated in the basement, and the part of it forming the back basement (which appears by the plan to contain sixteen single bedrooms) is appropriated to the worst cases. The incurables have not only a separate ward, but separate airing grounds also; and they do not, it is believed, intermix much with the other classes of patients.

From the foregoing details, it will be evident that Bethlem (in this respect differing from most of the other great public institutions for the insane in England) is pre-eminently and essentially an hospital for the treatment and cure of insanity, a fact which it will be material to bear in mind when the arrangements for supplying medical care and treatment to the patients are considered. It is to be observed, also, that the patients on the charitable foundation, although necessarily persons in straitened circumstances, are for the most part taken from the middle and educated classes; that comparatively few of them have earned their livelihood by manual or daily labour; that none of them belong to the pauper class; and that, from their previous habits and position in life, the great majority of them have been accustomed to observe the proprieties, and are fully capable of appreciating the comforts, of civilized society. [The tables annexed to the annual report show that upwards of five-sixths of the patients had received a superior, good, or moderately good education. This is, of course, exclusive of the criminal ward.]

Although, of late years, improvements have been introduced into the hospital, similar to those which for some time past have been in progress in other public lunatic asylums, the institution is still manifestly defective in many respects; one of the most striking defects being the inadequacy of the provision for the care and treatment of patients suffering under bodily diseases or infirmity.

The hospital at present possesses no sick-ward or infirmary. It has not the usual conveniences and appliances for sick or infirm patients, nor experienced nurses accustomed to the management of the sick.

It was stated by Dr. Wood, the resident apothecary, that occasionally when an epidemic broke out, he had placed those whom it attacked in a separate room, as far as possible removed from the main body of the inmates, but there is not, and there never has been, any systematic appropriation of apartments fitted up for the sick or infirm.

Amongst the extensive additions to the building which were commenced so long ago as the year 1838, a distinct "infirmary or sick-ward for each sex, with nurses' rooms attached," formed part of the plan publicly announced, although (for what reason does not appear) this has not, up to the present time, been carried out. The desirableness of such a provision is generally admitted by the medical officers.

It is suggested, indeed, by one of them, that as persons who, in the language of the rule, are "so sick as to require the attendance of nurses," as well as those who after admission fall into such a state, are thereupon rejected or discharged, as the case may be, the existence of a separate sick-ward or infirmary is of comparatively little moment.

We cannot admit the force of this argument. It has no application to the patients on the incurable and criminal lists, who constitute together about two-fifths of the entire number under treatment.

Patients once placed on the incurable list almost invariably remain in the hospital for the rest of their lives; and all, or nearly all of them, must in the ordinary course eventually die there. Not a few are persons advanced in years; and many become subject to and die under lingering and painful bodily diseases; among the more frequent causes assigned for these deaths, various maladies are specified, in which the most careful medical treatment and most sedulous nursing in a sick room would be peculiarly required. The same reasoning would apply in a considerable degree to the patients on the criminal list.

But even as regards the patients on the curable list, the argument will be found to have no real weight, when the facts are thoroughly examined.

It is to be observed that the rules regulating the admission and discharge of patients on that list have never been construed so stringently as to preclude the admission or retention of many insane persons, whose general health is bad.

The almost total absence of medical records renders it extremely difficult to ascertain the precise number of curable patients under treatment at any particular time, but enough may be collected from the annual reports to prove that there is always in Bethlem a large number of curable patients, who are in a bad state of bodily health, and who therefore require the best medical care and treatment. Of those who are admitted on the curable list, and are subsequently discharged uncured, after having remained the full period, about one-sixth appear to be in bad bodily health on admission, and it would not be unfair to assume that of those who are discharged cured from that list, the like proportion were on admission in a similar condition. At any rate, there is a legitimate presumption, that nearly all the curable patients who are prematurely sent away as disqualified, or by request (amounting on an average to about forty in a year), had fallen into bad bodily health prior to their discharge. Of course, until that condition becomes confirmed, and arrangements are made for their safe removal, they necessarily remain in the hospital, and during the intermediate time demand the best medical treatment and nursing. This was remarkably exemplified in the cases of the two female patients, A. M. and H. H., the circumstances of which will be hereafter fully detailed.

The bedding provided for the patients in the back basements, where such of the refractory patients as are wet or dirty sleep, is open to serious objection, and the state in which the females of that class are placed in bed is not merely most objectionable, but most discreditable. The bedding in that part of the ward for the most part consists of loose straw placed in a crib or trough-bedstead, and covered with a blanket, and upon this the patient is laid in a state of complete nudity. This practice, which has prevailed for years, has gone on without challenge from, and even (as is stated) without the knowledge of, the treasurer, the physicians, the resident apothecary, the matron, or any other of the responsible authorities of the institution. The existence of such a state of things, while it discloses a most reprehensible practice, at the same time proves that most culpable laxity must have prevailed in the internal supervision of the hospital.

In discussing the circumstances which render the fitting-up of an infirmary so desirable, we have already in a great measure anticipated the reasons why it is indispensable to the efficiency of the institution, that it should constantly command the services of a well-organized and efficient medical staff.

These reasons are mainly grounded on the peculiar character of Bethlem as an hospital for the treatment and cure of the insane; on the great number of persons who are annually admitted to its benefits; and on the unusually large proportion received in it, of cases in which active and judicious treatment (including under the term all remedial means, whether medical or moral) is likely to be useful.

When to the medical and moral treatment of the patients are added the multifarious duties comprised under the terms "general management and superintendence," duties which have been found by experience to be in general most conveniently attached to the office of the resident medical officer; it will readily be conceded, that in an hospital of the size and character of Bethlem, those labours are far too onerous to be adequately performed by a single individual. In point of fact, nearly the whole of the treatment (medical as well as moral), and much of the business of general supervision, are at present thrown upon Dr. Wood, who, as he candidly admits, has not been equal to the burthen; and the services of the visiting physicians (which apparently are almost entirely confined to examining the patients before admission and discharge, and to occasionally prescribing for them in cases of severe illness) occupy but a small portion of their time, and have not been always regularly or assiduously rendered. The result has been that the care of the patients is left far too much to the uncontrolled discretion of the attendants; that the keeping of the case-book is in a great measure neglected; that scarcely anything is known (or can now be ascertained from the records) respecting the past condition or treatment of patients who have left the hospital; and that various complaints have from time to time been made, the justice of which it is now extremely difficult to estimate, but which the evidence certainly bears out to some extent, of supineness and negligence on the part of the medical officers.

The apology urged by the resident apothecary in substance is, that an excessive amount of work has been thrown upon him, far beyond what he can satisfactorily accomplish; and it is impossible to deny that there is some force in the plea.

The proper discharge of such duties would, we conceive, furnish ample employment for at least two resident medical officers. The principal medical officer, in his capacity of general superintendent, should be invested with paramount authority within the hospital, and be responsible for the whole of its internal management; and to him the rest of the medical staff, the matron and the inferior officers and attendants, should all be subordinate. He might also be empowered, under the sanction and regulation of the sub-committee, to call in certain other eminent medical practitioners, in cases of difficulty or emergency.

The existing plan of having visiting physicians, between whom the medical care of the patients is now nominally divided, in our opinion encourages carelessness and destroys responsibility. Having regard to the amount of the salaries now paid to the medical staff, the scheme does not appear to be open to objection on the ground of its involving much additional expense.

By one of the printed rules of the hospital, it is provided that the resident apothecary shall keep a register, in which he shall enter the particulars of each case, as taken by the physicians, on the admission of the patients, with a weekly record (subject to the revision of the physicians) of the bodily and mental health of every curable patient, and of any change in their condition which from time to time may occur, together with the effect of all medicines administered; and also an account of all surgical operations whatever; and shall record every change in the bodily or mental health of any curable or criminal patients with the like precision; which register is to be signed, on the discharge or death of the patient, by the physician under whose care the patient has been placed.

Notwithstanding the explicit forms of this judicious regulation, it has not been enforced or attended to in practice. At present the case-book contains, with scarcely any exception, merely a brief summary of the condition of the patient at the respective periods of admission and discharge, without noticing any further particulars of the case, either as to its characteristic symptoms, or as to its subsequent phenomena and progress, so that it affords little or no really useful information. [In all registered hospitals and licensed houses subject to the regulations of the act 8 and 9 Vict., c. 100, medical case-books are regularly kept, pursuant to the 60th section of the statute. Bethlem Hospital, which is specially exempted from the operation of this act, forms an exception to the practice.] We could not learn that the defective character of the case-book, and the neglect of the rule in this respect, had ever been brought to the attention of the treasurer or sub-committee, by either of the visiting physicians, although the weekly record which it is directed to contain is declared by the same rule to be "subject to their revision."

The general superintendence of the hospital is carried on, as already stated, under the control of the treasurer and sub-committee, and the more immediate conduct of its internal economy and discipline is confided to the resident apothecary, the steward, and matron, under whose supervision the care of the patients in their respective wards is entrusted to attendants of both sexes.

For the instruction and guidance of these various functionaries, a body of printed rules and orders was some years ago issued, in which their several departments are to some extent defined, and their special duties prescribed.

Many of these rules and orders, however, have never been enforced, and have been allowed to become obsolete; others are ambiguous in their terms; others, again, having been found inconvenient, have been altered or rescinded, and new rules substituted in their place; and a considerable number of additional regulations and usages, more or less important, and some of them materially modifying the previously existing practice, have also been introduced at various times (occasionally on the sole authority of the treasurer), and have acquired the force of rules and orders, although they have not been reduced to writing, and have never formally received the sanction of the governing body. The consequence, as might be expected, is, that what purports to be now the code of rules and orders according to which the hospital is governed, is extremely loose and imperfect; that various important points are uncertain or unprovided for: and that its precise bearing and effect are not thoroughly understood even by those whose conduct it is intended to regulate.

It is not necessary to go through these defects in detail, but among the most important may be mentioned that the existing rules do not secure any system of careful inspection at irregular and uncertain intervals by the governing body or any of its members; that they vest (we think most injudiciously) in the matron the duty of classifying and employing the female patients, and determining in what wards they shall from time to time be placed, according to her sole discretion, and to the practical exclusion of all medical authority; and further, that they leave it uncertain on whom is now primarily imposed (whether the steward or the resident apothecary) the responsibility of seeing to the sufficiency of the bedding and clothing of the patients. On the whole, we feel ourselves warranted in saying, that in their present very anomalous and imperfect state, these rules and orders tend to produce confusion and mistakes; and that they are unfavourable to that cordial union and co-operation among the different officers of the establishment which are indispensable to the successful working of the institution.

Upon these grounds we are of opinion that the whole of the rules and orders, written and unwritten, according to which Bethlem is now professedly managed, ought to be carefully revised, and that they should then be issued in a complete form, and be thereafter rigidly enforced.

We now proceed to state the general result of the testimony of the several witnesses who were examined by us in reference to the subjects of our inquiry. The evidence was received by us on nine different days, namely, on the 7th, 10th, 17th, and 24th of July, the 14th August, the 8th and 22nd October, the 18th November, and the 4th December, 1851; the investigation having been extended and prolonged on account of fresh evidence having been from time to time tendered for our consideration, and by the fact that the testimony given upon one or more occasions rendered it expedient to pursue further some particular branch of the inquiry.

The witnesses who were examined were twenty-seven in number. Their names are as follows—viz., Mr. John Edward Johnson, treasurer of Bethlem Hospital; Dr. William Wood, resident apothecary of ditto; Henrietta Hunter, matron of ditto; Sarah Burrowes, Elizabeth Cliffin, Keziah Sanderson, nurses in Bethlem Hospital; Dr. Edward Thomas Monro, Sir Alexander Morrison, visiting physicians of ditto; Peter Northall Laurie, Esq, a governor of ditto; Mr. Thomas Joseph H., father of Miss H. H., lately a patient in ditto (dead); Mrs. Elinor W., lately a patient in ditto; Mr. Charles Taylor, surgeon; Elizabeth W., mother of Mary Elizabeth W., lately a patient in Bethlem Hospital; Mr. John Ogle Else, surgeon; Dr. P. R. Nesbitt, medical superintendent of the Northampton Lunatic Asylum; Dr. John Webster, a governor of Bethlem Hospital; Mr. Nathaniel Nicholls, steward of ditto; T. S. Capel, Esq., a governor of ditto; Miss Mary Elizabeth W., lately a patient in ditto; Wm. Marson, lately an attendant in ditto; John Welsh, ditto; Samuel H., brother of H., lately a patient in Bethlem Hospital (dead); Andrew Wentzell, friend of ditto; William Beech, lately an attendant in the Hospital; David Kidd, an attendant in ditto; Miss A. M., lately a patient in the hospital; Miss F. M., sister of ditto.

A copy of the short-hand writer's notes of the evidence given by the above-named witnesses before our board, accompanies this report.

This evidence appears mainly to embrace the following subjects—viz.

1. Miss A. M.'s case.
2. Miss H. H.'s case.
3. The cases of Mrs. Elinor W., Mrs. Mary Elizabeth W., and H.
4. The general government and management of the hospital.

We shall, in the first instance, draw your attention to the case of Miss A. M. (lately a patient in Bethlem Hospital), requesting at the same time a reference to the evidence itself, whenever our summary or statement of facts shall appear to require explanation.

It appears by the evidence laid before us, that Miss A. M. was admitted as a patient into Bethlem Hospital on Sunday, the 6th October 1850, and that she remained there till the 27th of the following December (a period of eleven weeks and five days), when she was discharged.

Miss M. F. (the patient's sister) states, that A. M., at the time of her admission was in weak bodily health, and had for some days refused her food; that she (M. F.) had visited her on the Friday after her admission (October 11), and again on the 4th of November. That on the first visit she perceived that A. M. had a black eye; that on the second visit she found that her sister had been removed from an upper storey into the basement-ward of the hospital, which it appears is the ward specially appropriated to dirty and refractory patients. That at this time A. M. complained of excessive cold, of having no sheets or blankets, and of sleeping on loose straw; and also of the coarse, violent, and abusive conduct of the nurses under whom she was placed. Subsequently, Miss F. M. understood from one of the nurses, that A. M. had become "a dirty patient."

In consequence of A. M. complaining repeatedly of cold, her sister on one occasion, took a warm jacket to the hospital for her, but the matron said that the patient "could not be allowed to wear it, as it was too smart."

Miss M. F. again visited her sister on the 3rd December, when she found her in a most feeble and emaciated state, complaining still of cold and of want of bed-covering; and also of a disorder in her bowels; and ultimately, in consequence of her sister's complaints of ill-treatment, and of her general personal appearance, Miss M. F. decided upon removing her, and in fact, did remove her from the hospital on the 17th of December. It appears that A. M., during almost the whole of her residence in the hospital, remained in the basement-ward.

The account given by Miss M. F. is corroborated by the patient's own evidence, taken on oath after she was perfectly recovered (both mentally and bodily), and when she had returned to her friends. From this it appears that she had always insufficient bedding during her residence in the basement-ward of the hospital, and sometimes none at all, only the straw, under which she crept for the sake of warmth. She had no night-clothing, but always slept quite naked, and suffered from cold, both night and day. She sometimes felt very ill, but her treatment by the nurses was very rough, and their conduct towards her was rude and coarse. She was covered, she says, at this time with sores, which she attributes to the kind of bed on which she lay.

According to the testimony of the resident apothecary and matron of Bethlem Hospital, and of the nurse who had the care of this patient in that establishment, it appears that at the time of Miss A. M.'s admission into the hospital her person was examined, according to custom, by one of the nurses there. At this time, according to every statement, there were "no bruises, wounds, or sores" on her body; and it is stated she was in moderate (but it is said below average) health. She was placed, in the first instance, in the receiving-ward, but was removed in about a fortnight (on the plea of her being a dirty patient) to the basement-ward of the hospital. This was done by the sole direction of the matron, who, it appears, has the power of removing and classifying the female patients, without the intervention of any of the medical officers.

During her residence in the basement, the matron and resident apothecary were respectively informed that there were marks on the patient's body, and that she had a bodily complaint (requiring great care and attention) and swollen legs; and it appears that being in a low state of health she was put upon sick diet; but neither the matron nor the resident apothecary seems to have particularly inspected her bodily condition, or to have examined the sores on her person; nor did either of them see her in bed, or know that she was put to bed naked upon loose straw.

During her residence in the hospital, it appears that she got into bad health and suffered under some bodily complaint, and that she was treated medically by the resident apothecary, and also by Dr. Monro, but unfortunately no record of any bodily disorder or of any medical treatment to which she was subjected, is to be found in the case-book, beyond the general fact of her being placed upon "sick diet."

It is shown by the testimony of two of the nurses having the care of the basement-wards, that this patient and others on the basement-floor (amounting together to about fifteen in number) were regularly put to bed entirely naked (without any linen or other covering on their persons), and that there was only a blanket interposed between them and the loose straw on which they slept, which blanket was sometimes removed by the patients in the course of the night. This usage does not appear to have been known either to the resident apothecary, or to the matron, or to either of the visiting physicians, or, in fact, to anybody connected with the establishment, with the exception only of some of the nurses, and of the patients themselves who were subjected to this unusual mode of treatment.

On Miss A. M. being removed, it is stated that she was examined by one of the nurses, and that her back was then "very red," supposed to be from her dirty habits, or perhaps, "from the rubbing of the straw;" that her legs were swollen; and that she had punctured wounds and some few bruises on her person.

She was removed from the hospital on the 27th December, and on the day of her removal her person was examined by her sister, who states that she found her back a mass of sores, with a large sore on each hip, her body emaciated, and her legs swollen; and it was also ascertained that she had a disorder in the bowels. Miss F. M. took her sister on the day of her removal to a house in Camberwell, and on the following morning took her to Northampton, for the purpose of placing her in the asylum there. Previously to her departure for Northampton, however, she was examined by Mr. Else, a surgeon, who had before certified as to her state of mind on the occasion of her admission into Bethlem Hospital. At that time Mr. Else says that Miss A. M.'s condition "was anything but good, but it was not so bad but that it might be amended."

On her leaving the hospital, he states that it was so bad that "he doubted her living twenty-four hours," and he strongly advised that she should not venture to take the journey to Northampton. It is right to say that Mr. Else did not examine the body of Miss A. M. at this time, but he understood that she had sores on her person.

On the 29th December, 1850, being the second day only after her removal from Bethlem Hospital, Miss A. M. was received into the Northampton Asylum. The following statement of her condition at that time, and of the remedies used in her case, is given by Dr. Nesbitt, the medical superintendent of that establishment.

"Her state upon admission was most deplorable; with an attenuated frame, her system was so enfeebled that she was unable to sit up. She had prolapsus of the uterus and anus, with great mucous discharges, and suffered severely from tenesmus. Her lower extremities were livid and œdematous, and their motion paralysed. On the hips and nates were a great many abrasions of the surface, varying in size from a millet-seed to a split-pea. She had also on different parts of her body copper-coloured eruptions; she was quite unconscious of the calls of nature, and her urine and fæces passed under her. The treatment pursued was in the first place to give her a comfortable bed, and to attend to her infirmities by frequent changes of linen; morphia, the hydriodate of potass, and the local applications of oak-bark decoction, with generous diet, enabled her to get up in the course of three weeks; and from that time to this, her course has been one of progressive improvement. She was also a quiet patient, and grateful for attention, and after one month's residence all her faulty habits entirely left her."

This statement was made in writing on the 11th May, 1851, in answer to our inquiries respecting the patient.

At this time Dr. Nisbett says, "Miss A. M's mental state is that of rationality, propriety, and decorum, and her bodily health is completely re-established. Her continuance here is more a matter of prudence and convenience than of necessity."

In a subsequent statement made in writing on the 14th of May, and being also in reply to some further questions proposed by this board, Dr. Nesbitt says that he has no longer any hesitation in giving his opinion that the sores and abrasions on Miss A. M.'s body were referable to neglect; and he expresses his opinion that the straw would produce such irritations on the surface of her body in connexion with her want of cleanliness. He observes that she had never evinced any propensity to pick her skin since she had been under his care.

In this statement Dr. Nesbitt further says that Miss A. M. had uniformly complained of having nothing but straw to sleep on, "without any kind of protection between her body and the straw;" of having had no clothes to cover her; and of the whole treatment exercised towards her in Bethlem Hospital having been harsh and coarse; and adds that this patient was remarkable for truthfulness, and was desirous of burying in oblivion that part of her life spent in the hospital.

Both of these written statements of Dr. Nesbitt were afterwards verified by oath.

On a subsequent occasion Dr. Nesbitt states in his evidence before us, that at the time of this patient's admission into the Northampton Hospital, "her hair was very much matted and entangled, and there was a great number of vermin in her head;" and he adds, that in his opinion the vermin and the sores were evidences of previous neglect, and that the sores appeared to be the result of her having lain upon straw.

Dr. Nesbitt's account of Miss A. M.'s rapid amendment under the mode of treatment pursued in the Northampton Asylum, is confirmed by the evidence of her sister, who visited her there. "A fortnight after her admission into the Northampton Asylum," Miss F. M. says that she "found her sister greatly improved in health, her habits of cleanliness returned, and her mind also greatly improved."

The result of the evidence as to this case appears to be—

1st. That this patient (A. M.) was admitted into Bethlem Hospital in moderate or weak health, but without any bruises, wounds, or sores on her person. That after remaining there nearly two months, she left the establishment in no respect apparently improved in mind, emaciated in person, in deplorable health, covered with sores or abrasions, with bad habits, suffering under more than one bodily disorder, and with her head full of vermin.

2nd. That on her removal to the Northampton Asylum, by affording her certain personal comforts, by generous diet, and by judicious medical and other treatment, she began almost immediately to improve; that in a fortnight she was manifestly better in every respect; that in a month her faulty habits entirely left her; that in rather more than four months her mental and bodily health were completely re-established; and that at the expiration of between five and six months she was discharged from the Northampton Asylum, and restored to her friends perfectly well in all respects.

3rd. That during her residence in Bethlem Hospital (besides acquiring the bad habits and bodily disorders above adverted to) she suffered almost continually from cold and insufficient bedding; that she was subjected to rough usage from the nurses; that notwithstanding the cold, she was allowed no night-clothing for her person, but slept naked on loose straw, which punctured her body, and produced sores; that neither the resident apothecary nor matron, nor the visiting physician whose patient she was, ever examined her person or saw her in bed; that the fact of her (and others) sleeping without night-clothing on loose straw, was (as is stated) altogether unknown to the matron and to all the medical officers and other authorities of the hospital; and that although she was in bad health, and was under medical treatment by Dr. Munro and the resident apothecary, there is no record thereof in the case-book of the hospital.

Another patient, to whose case our attention has been particularly called, is that of Miss H. H. This lady appears to have been admitted into Bethlem Hospital on the 4th of April last, to have been discharged on the 18th of the same month (after a residence therein of fourteen days only), and to have died in three days after her discharge.

At the time of her admission, it is stated by the medical practitioner who had previously attended her, that her limbs were firm, and that although thin, she had the average amount of flesh and strength for an invalid; but she occasionally refused her food, had two or three times been subjected to mechanical restraint, and for two days previously to her admission had fallen into dirty habits. Her father also states that on her admission her health was "pretty good," and that she was gaining flesh. In contradiction to this, the matron states that she was emaciated at this time, but she never personally examined her; and the resident apothecary says that she was "very emaciated;" but his case book does not set forth the fact. Sir Alexander Morison states that she was "very weak and thin."

About eight days after her admission, the patient's father states that he went to the hospital to inquire after her, and learned from Dr. Wood, the resident apothecary, and from one of the nurses, that his daughter (whom he did not then see) was somewhat improved, and that she took her food better.

About five or six days afterwards, feeling anxious about her, he wrote to Dr. Wood, begging, as he had been told that he could not be allowed to see his daughter for a month after her admission, to be informed should any bad symptoms arise. This letter was left at the hospital early in the morning of Friday, the 18th of April: at about half-past 2 o'clock on the same day he received an answer from Dr. Wood, informing him that his daughter was in a very precarious state; that it had been thought proper to discharge her; and that there was every reason to anticipate a fatal termination to her illness. She was accordingly discharged on the same day, and was removed by her father direct to his own house.

On her discharge, her condition, according to her father's account, was exceedingly changed for the worse; and according to the statement of his medical attendant, Mr. Taylor, "her appearance was much altered, being very emaciated, her bones almost protruding through her skin, and on her body were various wounds and bruises."

On the 21st of April, the day after her discharge from the hospital, she died. After her death, in consequence of some complaints made by the father, her person was inspected on the following day by Mr. Lawrence and Sir Alexander Morison (two of the medical officers of Bethlem Hospital); and a post-mortem examination was afterwards made by two surgeons, who, together with Dr. Wood, who came in before the examination was completed, signed a medical certificate, stating "that there was no evidence to show that she had sustained any injury which could in any way have hastened her death."

During H. H.'s residence in Bethlem Hospital, it appears that she and other female patients, fifteen in number, were regularly placed to sleep on loose straw, a blanket only being interposed between their bodies and the straw, and that they slept entirely naked, *i. e.*, without any linen or other covering on the body. That parts of H. H.'s person were very red on her admission, and that on her discharge there were various sores or punctures on her body, which seemed evidently to have been caused by the straw of her bed, into the midst of which she was in the habit of creeping.

It is stated that she was exceedingly feeble while in Bethlem Hospital, but that she had no sofa or easy chair, such as is used for infirm patients to sit on, during the day; that she was in the habit of crawling on her hands and knees on the floors of the institution, and that after her death wounds or abrasions were found on her knees and elbows.

During her residence in the hospital, H. H. was never seen in bed by the matron or by the resident apothecary, or by Sir Alexander Morison, whose patient she was. It is stated that none of them knew that she slept naked on loose straw—a practice which Sir Alexander Morison condemns altogether, considering that such bedding was particularly unfit for a weak or excoriating patient, such as H. H. in fact was. It is stated by Sir Alexander Morison, that he prescribed for her, and that she had sick diet. There is no record in the Case-Book of any medical treatment, with the single exception of an enema, which was given to the patient on the appearance of diarrhœa, on the day immediately preceding her discharge

After her discharge, H. H. (who appears then to have partially recovered her intellect) complained that bad language had been used towards her whilst she was in the hospital, and that she had been beaten. The medical attendant of the family is of opinion that she was ill-treated or harshly used there; and the patient's assertion as to the use of bad language and very rough treatment is corroborated by the evidence of Mrs. Elinor W., who was at that time a patient in the same ward with H. H., but who was afterwards discharged recovered.

In consequence of some complaints made by the father of this patient, as to his daughter's treatment whilst in the hospital, an inquiry into the case was instituted before a committee of governors, and some evidence was produced thereon; but the investigation (owing to the father not prosecuting the inquiry further) was not completed. The governors have not, it is said, signified otherwise than that they were satisfied with the facts that came out in the course of the investigation; the result, nevertheless, of which seems to be that a special order was issued by them that "every injury (however slight) and every bruise and accident should for the future be immediately reported."

The general mode of treating this patient whilst in Bethlem Hospital will sufficiently appear from the foregoing summary of her case.

There is so much conflict, however, between the statements made by the several witnesses as to her condition on admission into the hospital and on her discharge, and also as to the origin and character of the various wounds and abrasions on her body, that we think it better to refer to the accompanying evidence for information on these points. To this evidence we must also refer for an account of the singular circumstances under which the medical certificate, drawn out after the *post-mortem* examination took place, appears to have been framed and signed.

The other cases upon which evidence was volunteered before us were those of persons who had been patients in the hospital—namely, Mrs. Elinor W. and Miss Mary W., female patients who had been discharged, and who are still alive; and H., a male patient, who died almost immediately after his discharge.

The testimony of Mrs. Elinor W. relates, in a great measure, to the case of H. H., already detailed. It corroborates, however, also the statements made by other persons respecting the bedding and general accommodation of female patients in the basement-ward; the coarse manners of the nurses in that ward; their extremely rough usage of their patients; and the great want of regular supervision and inspection in that part of the establishment. It is right to state that the evidence of Elinor W. was taken some months after she had been discharged recovered, and that at that time she appeared to be perfectly well, and to be a trustworthy witness.

In the case of Miss Mary Elizabeth W., the testimony of her mother (Mrs.

Elizabeth W.) shows that this patient had various bruises on her person during her residence in the hospital: that whilst there she complained of ill-treatment and coarse conduct on the part of the nurses; and that she slept naked on loose straw, in the same manner as is set forth in the cases of A. M. and H. H. The truth of the mother's statement as to this patient's complaint of ill-usage, is strengthened by the evidence of Elinor W., to whom Mary Elizabeth W. made the same complaints whilst in the hospital. And it is remarkable that both these last-named witnesses concur in stating that Mary Elizabeth W., whilst in the hospital, expressed an intention of complaining to the governors of the ill-treatment that she had been subjected to, and that she was dissuaded from, or counteracted in, her intentions by the matron.

On examining this patient herself, it must be observed that the answers given by her to our questions did not bear out the statements of her mother and of Elinor W. Mary Elizabeth W.'s recollection and general intellect were, however, in so feeble and imperfect a state at the time of her appearing before us, that we did not think it proper to question her to the extent we should have done, had she appeared more competent to give evidence on the subjects of our inquiry.

The tenour of the evidence given as to the male patient H., is to the effect that he was a patient in the hospital for rather more than five weeks; that he was a strong muscular man, and free from bruises on his admission; that on his discharge he was exceedingly reduced in flesh, and had numerous bruises on his body; and that he complained (amongst other things) of ill-usage from the attendants, and particularly of his throat having been severely squeezed,—a circumstance that was corroborated by the appearance of his throat on his discharge, and is also corroborated by the evidence of John Welsh, who was a keeper at the time of H.'s residence in the institution, but who has since been discharged. This last-named patient (H.) died within a few days after leaving the hospital.

In concluding our report, we think it desirable, in order to bring the main results of our inquiry more definitely and clearly under your notice, to state our opinion:—

1st. That Miss A. M. during her residence in Bethlem Hospital was subjected to harsh and improper usage from the attend nts, and was neglected by the medical and other officers of the institution; that the bedding, clothing, and accommodation afforded to her were unfit and insufficient, and that thereby her health was materially injured, and her life put in peril.

2nd. That Miss H. H. during her residence in the hospital was subjected to harsh and improper usage from the attendants, and was neglected by the medical and other officers of the hospital, and that the bedding and other accommodation afforded to her were unfit and insufficient; and must, in our opinion, have materially injured her bodily health.

3rd. That Mrs. Elinor W., Miss Mary Elizabeth W., and H., were respectively subjected to harsh and improper usage from the attendants of the hospital, and that they were neglected therein.

4th. That the sub-committee, as at present constituted, is of too fluctuating and uncertain a character; and that the duties imposed on the members thereof are too much of a formal character, and that their investigations into the well-being and comfort of the patients, and the practical working of the institution, are insufficient, and are nowhere properly recorded.

5th. That the treasurer has, without due authority, taken from the resident medical officer the duties of classifying the female patients, of regulating their employment, and determining in which wards they shall be placed, and has injudiciously invested the matron with this power.

6th. That the duties of the visiting physicians are very imperfectly performed; that their visits to the hospital are of too formal and superficial a character; that they never inspect the hospital or visit their patients at night, and that their treatment of their respective patients is in a great measure left to the resident apothecary, whose own duties (if fully performed) are more than sufficient to occupy his time.

7th. That the duties of the resident apothecary are not fully performed; that his visits to the wards, his treatment of the patients, his supervision of the attendants, and his investigation into the patients' comforts are imperfect and insufficient; that his medical records are much neglected, aud that these neglects on his part are in some degree (but not altogether) owing to the duties of the visiting physicians having in a great measure fallen upon him.

8th. That the duties of the matron are very much neglected, and that she has very improperly vested in her the power of classifying the female patients, of employing them, and of determining in which wards they shall be placed, without the sanction of any of the medical officers.

9th. That the rules and orders of the hospital, as at present existing, are not well understood; that they tend to produce confusion and mistakes, and should be revised and amended.

10th. That the various case-books, and other records, which by the general rules of the institution are required to be kept, are, for the most part either only partially kept or are altogether neglected; and that it is most important that proper records should be kept for the purpose of ascertaining whether or not the patients of the hospital have been fairly and judiciously treated during their residence therein.

11th. That the present medical staff is insufficient, and that any new arrangement thereof should comprehend the services of (at least) two resident medical officers, one of whom should have paramount authority in the hospital, and should be responsible for the whole of the internal management thereof.

12th. That there are no infirmaries or proper accommodation for sick or infirm patients in the hospital; and that the existing practices (which do not appear to be founded on any rule of the hospital) of excluding the relatives and friends of patients from all access to the patients themselves for the space of one month after admission, and of sending away patients in cases where severe bodily illness supervenes upon their mental maladies after admission into the hospital, are objectionable, and should be reconsidered and modified.

Finally. We are of opinion that the management and condition of Bethlem Hospital are in many material respects most unsatisfactory, both in reference to the purposes for which it was founded, and the very large funds by which it is maintained.

On behalf of the Board,

(Signed) SHAFTESBURY, Chairman.

R. W. F. Lutwidge, Secretary.

